# Caregiver-reported evaluation for and diagnosis of fetal alcohol spectrum disorders in the United States

**DOI:** 10.1016/j.drugalcdep.2026.113055

**Published:** 2026-01-21

**Authors:** Nicholas P. Deputy, Ashleigh M. Kellerman, Amanda N. Dorsey, Clark H. Denny, Mary Kate Weber, Shawn A. Thomas, Jessica Jones, Shin Y. Kim, Jacquelyn Bertrand

**Affiliations:** a Division of Birth Defects and Infant Disorders, National Center on Birth Defects and Developmental Disabilities, Centers for Disease Control and Prevention, 4770 Buford Highway, Mailstop S106-3, Atlanta, GA 30341, United States; b US Public Health Service Commissioned Corps, 1101 Wootton Parkway, Rockville, MD 20852, United States; c Global Government Solutions (G2S) Corporation, 4372 N Loop 1064 W, Shavano Park, TX 78231, United States; d Epidemic Intelligence Service, Centers for Disease Control and Prevention, 1600 Clifton Road NE, Atlanta, GA 30329, United States; e Maternal and Child Health Bureau, Health Resources and Services Administration, 5600 Fishers Lane, Rockville, MD 20852, United States

**Keywords:** Fetal alcohol spectrum disorders, Public health surveillance, National Survey of Children’s Health, FASD evaluation, FASD diagnosis

## Abstract

**Background:**

In-person, active case ascertainment studies suggest the prevalence of children with fetal alcohol spectrum disorders (FASDs), lifelong disorders caused by prenatal alcohol exposure, in selected communities ranges from 11.3 to 71.4 per 1000 (including diagnosed and undiagnosed FASDs). National, population-based estimates of children with an FASD diagnosis and who are at different stages of a prototypical diagnostic process are limited.

**Methods:**

Data are from the 2022–2024 National Survey of Children’s Health (n = 160,640). We estimated the caregiver-reported prevalence of children who had ever been recommended for an FASD evaluation, received an FASD evaluation, and received an FASD diagnosis, overall and by demographic subgroups; Chi-square tests assessed differences by subgroup.

**Results:**

Based on caregiver report, 2.4 per 1000 children were recommended for an FASD evaluation, 3.1 per 1000 received an evaluation, and 2.0 per 1000 received an FASD diagnosis. Prevalence varied by several characteristics; for example, the prevalence of children with an FASD diagnosis was higher among those cared for by grandparents or other relations (10.8 per 1000, 95 % confidence interval [CI]: 7.2–16.0) than those cared for by two parents (1.2 per 1000, 95 % CI: 0.9–1.6). Of the 4.6 per 1000 children either recommended for an FASD evaluation, received an evaluation, or diagnosed with an FASD, 20.0 % had all three experiences.

**Conclusions:**

These nationally representative, caregiver-reported estimates for children with an FASD diagnosis are lower than those from active case ascertainment studies, suggesting efforts are needed to improve the screening, evaluation, and diagnosis process for children suspected of an FASD.

## 1. Introduction

Fetal alcohol spectrum disorders (FASDs) are a group of disorders, including fetal alcohol syndrome, caused by prenatal alcohol exposure (PAE). These disorders are characterized by combinations of central nervous system abnormalities, neurodevelopment and behavioral impairments, facial dysmorphia, growth deficits, and/or birth defects (Hoyme et al., 2016). There is no cure for FASDs; although persons with an FASD might require lifelong support and care, early intervention services can improve developmental and life outcomes (Turchi et al., 2018). Active-case ascertainment studies that utilize in-person assessments to identify children with and without a previously diagnosed FASD have found the prevalence of FASDs ranged from 11.3 to 71.4 per 1000 first grade children in selected communities in the United States (U.S.). Notably, only 1 % of children identified with an FASD in these studies had been previously diagnosed (May et al., 2018, 2021).

Estimating and describing the population of children with an FASD diagnosis is essential for understanding the magnitude and distribution of these disorders in the population, can inform the planning and development of services, and can provide a baseline estimate for efforts to improve diagnosis of FASDs. A study using healthcare claims databases found 0.12 per 1000 commercially insured and 0.61 per 1000 Medicaid insured children had an administratively reported FASD from 2015 to 2021 (Deputy et al., 2024). Although this study leveraged existing data and was less resource intensive than in-person studies, this study might underestimate the prevalence of children with an FASD diagnosis as studies of healthcare claims databases rely on a diagnosis being included on a claim and reported into healthcare databases, which might not consistently occur due to reimbursement or other reasons (Grosse et al., 2022). A recent study using data from the National Survey of Children’s Health (NSCH), an annual, nationally-representative, cross-sectional household survey that assesses various aspects of children’s health via caregiver report, found the prevalence of children with an FASD diagnosis was 2.1 per 1000 (Zang, et al., 2025). This study found prevalence varied across a limited number of demographic groups examined; however, a more comprehensive description of the prevalence of children with an FASD diagnosis by demographic characteristics is needed to inform public health activities.

Receipt of an FASD diagnosis (or diagnosis for another condition) is often the result of a multi-step process. Understanding the prevalence of children being recommended for and receiving an FASD evaluation – two steps in a prototypical FASD diagnostic process – can provide insight about FASD-informed care and areas for improvement (Hayes et al., 2023; Kent et al., 2023). However, no studies to date have captured the prevalence of children at different stages of the diagnostic process in a nationally representative U.S. sample.

In 2022, the NSCH assessed caregiver-reported FASD experiences for the first time, including recommendation for an FASD evaluation, receipt of an FASD evaluation, and diagnosis of an FASD. Caregivers are well suited to provide information about FASD evaluation and diagnosis as they typically raise concerns to a provider, follow-up on referrals for evaluation, and communicate diagnostic findings back to their medical home provider for treatment and care coordination of services. To provide an up-to-date and comprehensive description of FASD experiences in the U.S., we used pooled data from the 2022–2024 NSCH to estimate the caregiver-reported prevalence of children who were ever recommended for an FASD evaluation, who ever received an FASD evaluation, and who ever received an FASD diagnosis, overall and among demographic subgroups. We also examined the overlap in recommendation for an FASD evaluation, receipt of an FASD evaluation, and receipt of an FASD diagnosis to understand what proportion of children had more than one of these steps in the FASD evaluation and diagnostic process.

## 2. Materials and methods

### 2.1. Data source and sample

Data are from the 2022–2024 NSCH. The NSCH is an annual, nationally-representative, cross-sectional household survey funded and directed by the Health Resources and Services Administration, Maternal and Child Health Bureau and conducted by the U.S. Census Bureau; it is designed to provide national and state-level estimates for all non-institutionalized children aged 0–17 years in the U.S. The NSCH uses an address-based sampling approach to identify households with children and to select one child from the household to be the subject of the survey. During 2022–2024, children with special healthcare needs and children aged 0–5 years were oversampled. Age-specific surveys were administered to caregivers in English and Spanish in a paper or online format and assessed a range of child and family demographics, neighborhood and social contexts, mental and physical health (including diagnosed health conditions), and experiences related to healthcare accessibility. The NSCH interview completion rates (i.e., the proportion of households who completed the survey, among those that started the survey) were 78.5 %, 76.3 %, and 78.5 % in 2022, 2023, and 2024, respectively, and the overall response rates were 39.1 %, 35.8 % and 36.3 % in 2022, 2023, and 2024, respectively. Overall, 54,103 surveys were completed in 2022, 55,162 were completed in 2023, and 51,375 were completed in 2024, resulting in a pooled sample size of 160,640. Further information about the administration of the NSCH is available elsewhere (U.S. Census Bureau, 2023, U.S. Census Bureau, 2024, U.S. Census Bureau, 2025).

### 2.2. Variables

Three FASD-related questions were included for the first time on the 2022 NSCH; questions were cognitively tested to assess clarity and comprehension by members of the general U.S. population with children aged 0–17 and by families caring for a child with an FASD. On the survey, caregivers reported whether their child ever 1) was recommended for an FASD evaluation by a healthcare provider or educator, 2) had an FASD evaluation, and 3) received an FASD diagnosis by a healthcare provider. To fully capture potential patterns in an FASD evaluation and diagnostic process, skip patterns were not implemented such that all questions were presented to caregivers, regardless of previous responses to any FASD-related question.

Demographic information reported by caregivers was categorized based on sample size and consistent with previous NSCH reports (Ghandour et al., 2022); these included child’s age at the time of the survey (0–5, 6–11, or 12–17 years), child’s sex (male or female), child’s race and ethnicity (considered as a social construct, including Hispanic, non-Hispanic Black, non-Hispanic White, non-Hispanic American Indian or Alaska Native, or Other [collapsed due to limited sample sizes and including non-Hispanic Asian, Non-Hispanic Native Hawaiian and other Pacific Islander, and non-Hispanic two or more races]), primary household language (English or non-English), family structure (two parents [married or unmarried, including adoptive parents], single parent, grandparent or other relation [including foster parents]), highest level of caregiver education (high school or less, or some college or greater), child’s insurance status and type (public only [government assistance], private only [privately purchased, including through a marketplace or employer, or TRICARE], private and public, or none), household income relative to the federal poverty level (less than 100 %, 100 %–199 %, 200 %–399 %, 400 % or more), caregiver military status (one or both caregivers currently or formerly on active duty, or no active duty service), geographic region (Northeast, Midwest, South, West), and rurality (metropolitan statistical area residence or not). We also created a single composite measure assessing whether a child ever had a history of one or more other mental, behavioral, or developmental disorders, consistent with previous studies (Claussen et al., 2023); these included anxiety problems, depression, behavior or conduct problems, developmental delay, intellectual disability, speech or other language disorder, learning disability, Tourette syndrome, autism spectrum disorder, and attention-deficit/hyperactivity disorder (ADHD).

### 2.3. Analysis

We estimated weighted prevalence (per 1000 children) and associated 95 % confidence intervals (CI) for caregiver-reported recommendation for an FASD evaluation, receipt of an FASD evaluation, and FASD diagnosis, overall and by demographic characteristics. Chi-square tests were used to assess differences in the prevalence of FASD evaluation recommendation, evaluation receipt, and FASD diagnosis by characteristics; statistical significance was considered *p* < 0.05. To capture steps in the FASD evaluation and diagnostic process, among children who had either an FASD evaluation recommended, received an FASD evaluation, or had an FASD diagnosis, we estimated the proportion of children with different combinations of these experiences reported. In addition, because neurodevelopmental characteristics of FASDs commonly overlap with other developmental disorders, we conducted a post hoc analysis to assess what proportion of children who received an FASD evaluation, but did not have an FASD diagnosis, had a history of another mental, behavioral, or developmental disorder diagnosis.

All analyses were weighted to account for the probability of selection, nonresponse, and underlying demographic distribution of the target population to generate national estimates of non-institutionalized children aged 0–17 years in the U.S. The 2022 – 2024 NSCH public use datasets included imputed data for selected variables with missing information. Specifically, child sex, race, and ethnicity were imputed using hot-deck methods, missing education was imputed using a single imputation sequential regression method, and federal poverty level was multiply imputed using sequential imputation methods; in this analysis, estimates incorporating federal poverty level were calculated separately for each implicate and then combined using standard procedures (U.S. Census Bureau, 2023). Relative standard errors (RSE) were calculated for all estimates (standard error of an estimate / estimate) to evaluate the precision of estimates; imprecise estimates (RSE >30 %) are indicated as such. Analyses were conducted using SAS-callable SUDAAN version 11.0.1 (RTI International) to account for the complex survey design.

## 3. Results

Overall, according to caregiver report, 2.4 per 1000 children (95 % CI: 1.9–3.1; unweighted n = 339) were recommended for an FASD evaluation, 3.1 per 1000 (95 % CI: 2.5–3.9; unweighted n = 521) received an evaluation, and 2.0 per 1000 (95 % CI: 1.5–2.7; unweighted n = 329) received an FASD diagnosis, at some point during their childhood (Table 1). Caregiver-reported prevalence of the three FASD experiences varied by several characteristics; notable patterns are described below. For example, by race and ethnicity, recommendation for an evaluation and receipt of a diagnosis were higher among non-Hispanic American Indian or Alaskan Native children (28.9 per 1000 [95 % CI: 10.3–78.0]) and 30.2 per 1000 [95 % CI: 11.0–79.8], respectively) than non-Hispanic white children (1.2 per 1000 [95 % CI: 0.9–1.6) and 1.2 per 1000 [95 % CI: 0.9–1.5], respectively), though estimates for non-Hispanic American Indian or Alaskan Native children were imprecise (RSE >30 %). Specific to family structure, recommendation for an evaluation, receipt of an evaluation, and receipt of a diagnosis were higher among children cared for by a grandparent or other relation (10.6 per 1000 [95 % CI: 6.8–16.5], 9.6 per 1000 [95 % CI: 6.2–14.9], and 10.8 per 1000 [95 % CI: 7.2–16.0), respectively) than those cared for by two parents (1.3 per 1000 [95 % CI: 1.0–1.7], 2.3 per 1000 [95 % CI: 1.9–2.8], and 1.2 per 1000 [95 % CI: 0.9–1.6), respectively). Recommendation for an evaluation, receipt of an evaluation, and receipt of a diagnosis were higher among children with public insurance only (4.5 per 1000 [95 % CI: 2.9–6.9], 4.9 per 1000 [95 % CI: 3.3–7.4], and 4.1 per 1000 [95 % CI: 2.6–6.4], respectively) than those with private insurance only (0.6 per 1000 [95 % CI: 0.5–0.9), 1.6 per 1000 [95 % CI: 1.3–2.1], and 0.8 per 1000 [95 % CI: 0.5–1.2], respectively). Similarly, recommendation for an evaluation, receipt of an evaluation, and receipt of a diagnosis were higher among children who ever had a history of one or more mental, behavioral, or developmental disorders (6.8 per 1000 [95 % CI: 5.5–8.4], 7.9 per 1000 [95 % CI: 6.5–9.6], and 5.7 per 1000 [95 % CI: 4.6–7.1], respectively) than those without such a disorder (0.8 per 1000 [95 % CI: 0.3–1.9], 1.4 per 1000 [95 % CI: 0.8–2.5], and 0.7 per 1000 [95 % CI: 0.3–1.9], respectively), though some estimates for children without a history of mental, behavioral, or developmental disorders were imprecise (RSE >30 %).

Overall, 4.6 per 1000 children (95 % CI: 3.9–5.4; unweighted n = 715) had either an FASD evaluation recommended, received an FASD evaluation, or had an FASD diagnosis. Among this subgroup, 19.7 % were recommended for an evaluation but did not receive an evaluation or diagnosis, 8.5 % were recommended for an evaluation and received an evaluation, but were not diagnosed with an FASD, and 20.0 % were recommended for an evaluation, received an evaluation, and were diagnosed with an FASD. The remaining children had a different combination of FASD evaluation and diagnosis experiences; for example, 10.5 % had an FASD diagnosis, but were not recommended for or received an evaluation (Table 2). In post hoc analysis, among children who received an evaluation but were not diagnosed (regardless of recommendation; n = 346), 62.2 % also had a history of one or more mental, behavioral, or developmental disorders (data not shown).

## 4. Discussion

In this nationally representative sample, the caregiver-reported prevalence of children with an FASD diagnosis was 2.0 per 1000 children; furthermore, 2.4 per 1000 children were recommended for an FASD evaluation and 3.1 per 1000 received an evaluation. Although estimates of children with an FASD diagnosis are higher than those from healthcare claims databases, they likely underestimate the entire population of children with FASDs (i.e., those with and without an FASD diagnosis). However, patterns in caregiver-reported prevalence of FASD diagnosis by demographic characteristics align with previous reports that have found higher prevalence among American Indian children (Popova et al., 2019), children with public insurance (Deputy et al., 2024), and children under the care of a grandparent or other relative (Lange et al., 2017; Popova et al., 2019). Assessing the prevalence of children with an FASD diagnosis, overall and by demographic characteristics, is essential for understanding the magnitude and distribution of these disorders in the population, gauging current service needs, identifying populations requiring additional support services, and can serve to establish a baseline to evaluate efforts focusing on increasing identification of children with FASDs.

Prevalence estimates for children with FASDs vary depending on study methodology. Specifically, this study found 2.0 per 1000 children had an FASD diagnosis based on caregiver report, which is consistent with estimates from a previous study that used 2022–2023 NSCH data (Zang et al., 2025). These caregiver-reported estimates are higher than a study of healthcare claims databases that found the administratively reported prevalence of FASDs was 0.12 per 1000 among children with commercial healthcare plans and 0.61 per 1000 among children enrolled in Medicaid during 2015–2021. Caregiver-reported prevalence of children with an FASD diagnosis might be higher than administratively reported prevalence due to the limited number of diagnosis codes that apply to FASDs and because there are no validated algorithms to identify children with FASDs in administrative databases (O’Donnell et al., 2022). Both caregiver-reported and administratively-reported prevalence of children with an FASD diagnosis are lower than those from active case ascertainment studies, which estimate the prevalence of FASDs range from 11.3 to 71.4 per 1000 school-age children (May et al., 2018, 2021). Active case ascertainment studies employ in-person assessments in selected community samples to evaluate FASD-related characteristics in children (e.g., dysmorphology, PAE) and can identify children who have and have not been previously diagnosed with an FASD, which might better approximate the true, underlying population of children with FASDs and likely explains higher estimates compared to other methods. Despite the NSCH underestimating the true population of children with an FASD, the nationally representative and ongoing nature of these data suggest it might serve as an important data source for public health surveillance activities focusing on children with an FASD diagnosis.

The sizeable difference between the prevalence of children with an FASD diagnosis from this study and the underlying prevalence of children with an FASD (i.e., those with and without a previously diagnosed FASD) from active case ascertainment studies highlight challenges with identifying and diagnosing children with FASDs. Indeed, one study found 80 % of youth with an FASD in a child welfare agency had not been previously diagnosed (Chasnoff et al., 2015). Previous studies have found that underdiagnosis of FASDs is likely related to social stigma associated with assigning a diagnosis to a family, stigma and difficulty around establishing a history of PAE, distinguishing diagnostic characteristics that have similar developmental profiles to other mental or behavioral disorders (e.g., ADHD), and limited diagnostic resources in communities (Benz et al., 2009; Popova et al., 2023; Wozniak et al., 2019). Identifying children with an FASD is important as it facilitates appropriate treatment and care and may optimize health and developmental outcomes. Strategies such as universal screening of PAE in pediatric settings may improve identification of children with FASDs, but studies suggest providers do not routinely assess PAE history for pediatric patients (Dunkley et al., 2025). Importantly, the American Academy of Pediatrics provides resources and trainings that promote universal screening of PAE, increase awareness of FASD diagnostic characteristics, and facilitate the FASD screening, assessment, and diagnosis process (American Academy of Pediatrics, 2017, 2023).

Examining the overlap of FASD-related experiences might provide insight into pathways through care for children suspected of an FASD. For example, among children who were either recommended for an evaluation, received an evaluation, or diagnosed with an FASD, 20.0 % experienced all three steps, which might reflect a prototypical diagnostic process. However, other patterns in FASD experiences were observed, including 10.5 % of children receiving an FASD diagnosis, but not reporting a recommendation for an evaluation and not reporting receipt of an evaluation. These patterns might reflect environments in which awareness of FASDs among providers is limited, referral or evaluation services are unavailable, or may reflect broader variability in diagnostic processes; these patterns might also reflect potential disconnects in the FASD evaluation and diagnostic process. Indeed, studies have identified provider-oriented challenges to FASD-informed care, such as limited training in identifying FASD signs and symptoms that contribute to delayed, incorrect, or lack of diagnosis, limited clinical capacity for multidisciplinary approaches to evaluation, and concerns about making a traditionally stigmatizing diagnosis (Abdul-Rahman et al., 2023; Brown et al., 2019; Dugas et al., 2022). Equally important are caregiver-oriented challenges that may impact pathways through care, such as lack of resources to navigate the diagnostic process, stigma, and changes in guardianship that might limit historical knowledge about a child’s health or care (Chamberlain et al., 2017; Morehouse et al., 2023). Moreover, for children with a history of ever having another mental, behavioral, or developmental disorder, the focus on FASD-specific concerns may be overshadowed by a more pressing and/or less stigmatized health concern (e.g., ADHD, autism), and, in our study, we observed a higher prevalence of FASD evaluation recommendation, evaluation receipt, and diagnosis among those with a history of another mental, behavioral, or developmental disorder. Future analyses are needed to explore pathways through care and how factors, including co-occurring disorders, might impact these processes.

This study is subject to limitations. Caregiver-reported prevalence of children with an FASD diagnosis underestimates the true population of children with FASDs due to underdiagnosis of these disorders in the general population. Small sample sizes reduced the precision of prevalence estimates among subgroups of interest. Although questions assessing recommendation for and receipt of an FASD evaluation provide insight into care pathways, they do not capture all potential steps or pathways to an FASD diagnosis, nor provide information on why an FASD diagnosis was ultimately not received. Ages at FASD evaluation and diagnosis were not captured, which limits insight into temporality of these experiences; furthermore, timing of FASD experiences relative to the survey administration might result in lengthy recall times that might influence reporting of these experiences. All FASD-related questions on the NSCH underwent cognitive testing; however, *FASD evaluation* was not explicitly defined on the survey, which might introduce ambiguity and contribute to misreporting or item nonresponse. Although the NSCH captures detailed information about caregivers, it does not distinguish biological and adoptive parents, and it is conceivable that caregiver underreporting may be attributed to changes in primary guardianship (e.g., for children cared for by adoptive or foster parents) that limits historical context about prior medical experiences; additionally, we were unable to distinguish grandparents from other relations due to limited sample sizes. Finally, the survey is based on caregiver report, and stigma associated with FASDs might have influenced reporting of these experiences, resulting in misclassification.

## 5. Conclusion

In summary, these caregiver-reported estimates represent the most recent national, population-based description of FASD evaluation and diagnosis among U.S. children. Findings from this study can complement those from active case ascertainment and healthcare claims studies by describing the population of children diagnosed with an FASD among a national sample. Furthermore, the availability of FASD-related questions on the NSCH allows for continual assessments to evaluate trends over time and detailed analyses among subgroups of interest. The sizeable difference between the prevalence of children with an FASD diagnosis described here and prevalence of children with and without an FASD diagnosis from active case ascertainment studies – in addition to the non-direct pathways through care described here – underscore the need to improve the screening, evaluation, and diagnostic process to ensure children with FASDs are evaluated and diagnosed in a timely manner to optimize outcomes.

## Figures and Tables

**Table 1:** Prevalence of Caregiver-Reported Recommendation for FASD Evaluation, Receipt of an FASD Evaluation, and FASD Diagnosis, Overall and by Demographic Characteristics – National Survey of Children’s Health, 2022 – 2024.

			FASD Evaluation Recommended (n = 339)		FASD Evaluation Received (n = 521)		FASD Diagnosis (n = 329)	
					
	Unweighted Sample Size^a^	Weighted % (95 % CI)	Weighted Prevalence per 1000 (95 % CI)	P-Value	Weighted Prevalence per 1000 (95 % CI)	P-Value	Weighted Prevalence per 1000 (95 % CI)	P-Value
								
**Overall**	160,640		2.4 (1.9–3.1)		3.1 (2.5–3.9)		2.0 (1.5–2.7)	
**Child’s Age**				0.9541		0.7532		0.3267
0–5 years	55,106	30.6 (30.2–31.1)	2.2 (1.0–4.7)*		2.6 (1.3–5.2)*		1.9 (0.8–4.5)*	
6–11 years	46,149	33.4 (33.0–33.9)	2.5 (1.8–3.5)		3.2 (2.4–4.3)		1.7 (1.1–2.5)	
12–17 years	59,385	35.9 (35.5–36.4)	2.5 (1.9–3.2)		3.4 (2.7–4.2)		2.4 (1.9–3.0)	
**Child Sex**				0.2647		0.4601		0.1010
Male	82,615	51.2 (50.7–51.7)	2.8 (1.8–4.1)		3.3 (2.4–4.7)		2.5 (1.6–3.8)	
Female	78,025	48.8 (48.3–49.3)	2.0 (1.6–2.7)		2.8 (2.2–3.7)		1.5 (1.1–2.0)	
**Child’s Race and Ethnicity**				**0.0006**		0.1778		**0.0151**
Hispanic	24,351	27.2 (26.7–27.7)	3.2 (1.8–5.9)*		3.3 (1.8–6.0)*		2.5 (1.2–5.2) *	
Non-Hispanic Black	9772	12.5 (12.2–12.9)	4.0 (2.4–6.6)		3.9 (2.3–6.8)		2.4 (1.4–4.1)	
Non-Hispanic White	103,606	47.4 (46.9–47.9)	1.2 (0.9–1.6)		2.4 (2.0–3.0)		1.2 (0.9–1.5)	
Non-Hispanic American Indian or Alaska Native	846	0.6 (0.5–0.7)	28.9 (10.3–78.0)*		31.5 (11.9–80.4)*		30.2 (11.0–79.8)*	
Other^c^	22,065	12.3 (12.0–12.5)	2.5 (1.7–3.6)		3.0 (2.2–4.2)		2.4 (1.5–3.9)	
**Household Language**				0.3669		0.8890		0.6512
English	146,084	84.1 (83.6–84.5)	2.1 (1.7–2.7)		3.1 (2.6–3.7)		1.9 (1.5–2.3)	
Non-English	13,710	15.9 (15.5–16.4)	3.7 (1.5–8.9)*		2.9 (0.9–8.7)*		2.6 (0.8–8.5)*	
**Family Structure**				**< 0.0001**		**0.0012**		**< 0.0001**
Two Parents (Married or Unmarried)^d^	120,662	72.2 (71.7–72.7)	1.3 (1.0–1.7)		2.3 (1.9–2.8)		1.2 (0.9–1.6)	
Single Parent	30,098	23.4 (22.9–23.8)	3.1 (2.2–4.4)		3.2 (2.3–4.6)		1.9 (1.3–2.6)	
Grandparent or Other Relation^e^	5384	4.4 (4.2–4.6)	10.6 (6.8–16.5)		9.6 (6.2–14.9)		10.8 (7.2–16.0)	
**Highest Level of Caregiver Education**				0.0793		0.5595		0.4746
High School or Less	24,024	27.2 (26.7–27.7)	3.8 (2.2–6.5)		3.6 (2.0–6.4)*		2.5 (1.2–5.2)*	
Some College or Greater	136,616	72.8 (72.3–73.3)	1.9 (1.5–2.4)		2.9 (2.4–3.5)		1.8 (1.4–2.3)	
**Child’s Insurance Type**				**< 0.0001**		**< 0.0001**		**0.0002**
Public Only (Government Assistance)	33,207	28.9 (28.5–29.4)	4.5 (2.9–6.9)		4.9 (3.3–7.4)		4.1 (2.6–6.4)	
Private Only (Privately Purchased, Including through ACA Marketplace, Employer, or TRICARE)	109,754	58.4 (57.9–58.9)	0.6 (0.5–0.9)		1.6 (1.3–2.1)		0.8 (0.5–1.2)	
Private and Public	7477	5.3 (5.1–5.6)	10.4 (6.5–16.7)		10.8 (6.8–17.0)		5.3 (2.7–10.1)*	
Not Insured	7260	7.3 (7.0–7.6)	3.2 (1.6–6.4)*		2.3 (0.7–7.1) *		1.5 (0.7–3.4)*	
**Household Income** ^f^				0.0511		0.4693		0.3882
< 100 % of Federal Poverty Level	19,380	17.7 (17.2–18.1)	4.4 (2.2–8.9)*		4.5 (2.2–9.3)*		3.0 (1.2–7.6)*	
100% – 199% of Federal Poverty Level	24,726	19.8 (19.3–20.3)	3.7 (1.8–7.3)*		3.6 (1.8–7.4)*		2.4 (1.0–6.0)*	
200 % – 399 % of Federal Poverty Level	46,498	28.2 (27.7–28.7)	1.7 (0.7–4.0)*		2.1 (1.2–4.0)		1.9 (0.9–4.0)*	
> = 400 % of Federal Poverty Level	70,037	34.4 (33.9–34.8)	1.3 (0.8–2.1)		2.8 (2.1–3.9)		1.4 (0.9–2.2)	
**Caregiver Military Status**				0.1262		0.0676		0.0906
One or Both Caregivers Currently or Formerly on Active Duty (Including Training in Reserves/National Guard)	15,708	8.9 (8.7–9.2)	3.9 (2.1–7.0)*		4.8 (2.9–7.8)		3.4 (1.8–6.4)*	
Neither Caregiver Served on Active Duty	139,673	91.1 (90.8–91.3)	2.0 (1.7–2.5)		2.6 (2.2–3.0)		1.6 (1.3–1.9)	
**Geographic Region**				0.3778		**0.0426**		0.1560
Northeast	27,160	15.8 (15.6–16.1)	1.9 (1.1–3.6)*		2.2 (1.5–3.4)		1.4 (0.8–2.4)	
Midwest	40,002	20.9 (20.7–21.1)	3.1 (2.2–4.4)		4.5 (3.4–5.9)		2.4 (1.8–3.3)	
South	45,204	39.5 (39.2–39.9)	2.0 (1.0–3.9)*		2.8 (1.6–4.7)		1.7 (0.8–3.7)*	
West	48,274	23.7 (23.5–24.0)	2.8 (2.1–3.7)		3.0 (2.3–3.9)		2.5 (1.8–3.5)	
**Metropolitan Statistical Area Status**				0.4204		0.0756		**0.0344**
Metropolitan Statistical Area	119,687	87.5 (87.2–87.8)	2.3 (1.7–3.2)		2.9 (2.2–3.8)		1.8 (1.2–2.6)	
Not Metropolitan Statistical Area	25,577	12.5 (12.2–12.8)	2.9 (1.9–4.5)		4.4 (3.2–6.0)		3.1 (2.2–4.4)	
**Diagnosis of Mental, Behavioral, or Developmental Disorders** ^g^				**< 0.0001**		**< 0.0001**		**< 0.0001**
No	110,174	72.8 (72.4–73.2)	0.8 (0.3–1.9)*		1.4 (0.8–2.5)		0.7 (0.3–1.9)*	
Yes	46,107	27.2 (26.8–27.6)	6.8 (5.5–8.4)		7.9 (6.5–9.6)		5.7 (4.6–7.1)	

Abbreviations: ACA = Affordable Care Act; FASD = Fetal Alcohol Spectrum Disorders.

a Sample sizes by demographic subgroups might not sum to overall sample due to missing data

b Estimate is imprecise (relative standard error >30 %); interpret estimate with caution.

c Includes non-Hispanic Asian, Non-Hispanic Native Hawaiian and other Pacific Islander, and non-Hispanic two or more races.

d Includes adoptive parents.

e Includes foster parents.

f Unweighted sample sizes across Household Income categories sum to greater than the overall sample size due to rounding during multiple imputation summarizing procedures.

g Includes the following conditions: anxiety problems, depression, behavior or conduct problems, developmental delay, intellectual disability, speech or other language disorder, learning disability, Tourette syndrome, autism spectrum disorder, attention-deficit/hyperactivity disorder.

**Table 2:** Patterns in Recommendation for an FASD Evaluation, Receipt of an FASD Evaluation, and Receipt of an FASD diagnoses, among Children with One or More FASD-Related Experiences Reported (n = 715).

Recommended for an Evaluation	Received an Evaluation	Received a Diagnosis	Weighted Percent^a^ (95 % CI)

**✓**			19.7 (14.4–26.4)
**✓**	**✓**		8.5 (5.3–13.3)
**✓**	**✓**	**✓**	20.0 (11.9–31.6)
		**✓**	10.5 (7.1–15.1)
	**✓**		36.3 (29.1–44.1)
	**✓**	**✓**	2.1 (1.2–3.8)^b^
**✓**		**✓**	2.9 (1.6–5.2)

a Weighted percentages do not sum to 100 % due to rounding.

b Estimate is imprecise (relative standard error >30 %); interpret estimate with caution.
